# Supplementary material for: Proteotranscriptomic Profiling of the Toxic Mucus of Kulikovia alborostrata (Pilidiophora, Nemertea)
Source: Toxins (Basel). 2024 Dec 26;17(1):5. doi: 10.3390/toxins17010005 (PMC11768458; doi:10.3390/toxins17010005)
Supplement: Supplementary file 1 [file toxins-17-00005-s001.zip › Supplementary File S4.zip!/ORF 001200 LINALB/1362d454f90accc2/dom_summary.html]

Phyre 2 Results for ORF\_001200\_LINALB

|  |  |
| --- | --- |
| Email | vas9i-kz@mail.ru |
| Description | ORF\_001200\_LINALB |
| Date | Fri Apr 7 03:17:43 BST 2023 || Unique Job ID | 1362d454f90accc2 |

|  |  |
| --- | --- |
| Domain analysis |  |

| Rank | Aligned region |
| --- | --- |

|  |  |
| --- | --- |
| 1 | --- \| c2f2jA\_ \| --- |
| 2 | --- \| d1n1ua\_ \| --- |
| 3 | --- \| c1n1uA\_ \| --- |
| 4 | --- \| c7k7xA\_ \| --- |
| 5 | --- \| c4ttoA\_ \| --- |
| 6 | --- \| c4ttmA\_ \| --- |
| 7 | --- \| d1nb1a\_ \| --- |
| 8 | --- \| c1nb1A\_ \| --- |
| 9 | --- \| c2khbA\_ \| --- |
| 10 | --- \| c7lhcA\_ \| --- |
| 11 | --- \| c2mn1A\_ \| --- |
| 12 | --- \| c4ttnA\_ \| --- |
| 13 | --- \| d1pt4a\_ \| --- |
| 14 | --- \| c2kukA\_ \| --- |
| 15 | --- \| c7rfaA\_ \| --- |
| 16 | --- \| c1jjzA\_ \| --- |
| 17 | --- \| c1k48A\_ \| --- |
| 18 | --- \| c2f2iA\_ \| --- |
| 19 | --- \| c2jwmA\_ \| --- |
| 20 | --- \| c3e4hA\_ \| --- |
| 21 | --- \| --- \| --- |
